# 1-{[5-(4-Chloro­phen­yl)-1-(4-fluoro­phen­yl)-1*H*-pyrazol-3-yl]carbon­yl]}piperidin-4-one

**DOI:** 10.1107/S1600536810047215

**Published:** 2010-11-20

**Authors:** Tara Shahani, Hoong-Kun Fun, R. Venkat Ragavan, V. Vijayakumar, M. Venkatesh

**Affiliations:** a X-ray Crystallography Unit, School of Physics, Universiti Sains Malaysia, 11800 USM, Penang, Malaysia; b Organic Chemistry Division, School of Advanced Sciences, VIT University, Vellore 632 014, India

## Abstract

In the title compound, C_21_H_17_ClFN_3_O_2_, the 1*H*-pyrazole ring makes dihedral angles of 36.73 (7), 18.73 (7) and 60.88 (8)°, respectively, with the mean planes of the chloro­phenyl, 4-oxo­piperidine and fluoro­phenyl rings. The mol­ecular structure is stabilized by an intra­molecular C—H⋯N hydrogen bond, which forms an *S*(6) ring motif. In the crystal, inter­molecular C—H⋯O hydrogen bonds link mol­ecules into chains along [101]. In addition, inter­molecular C—H⋯F hydrogen bonds with an *R*_2_^1^(7) ring motif connect neighbouring chains into layers parallel to the *ac* plane.

## Related literature

For pyrazole derivatives and their microbial activities, see: Ragavan *et al.* (2009 ▶, 2010 ▶). For a related structure, see: Shahani *et al.* (2010 ▶). For ring conformations, see: Cremer & Pople (1975 ▶). For hydrogen-bond motifs, see: Bernstein *et al.* (1995 ▶). For bond-length data, see: Allen *et al.* (1987 ▶). For the stability of the temperature controller used in the data collection, see: Cosier & Glazer (1986 ▶).
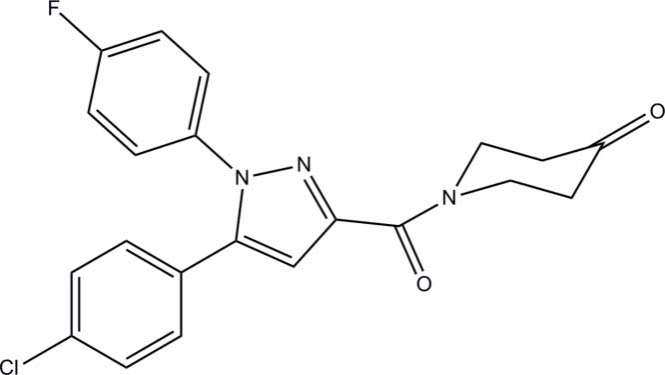


## Experimental

### 

#### Crystal data

C_21_H_17_ClFN_3_O_2_
*M* *_r_* = 397.83
Triclinic, 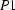

*a* = 6.0341 (2) Å
*b* = 8.2500 (3) Å
*c* = 10.2448 (3) Å
α = 108.837 (1)°
β = 104.782 (1)°
γ = 92.792 (1)°
*V* = 461.90 (3) Å^3^
*Z* = 1
Mo *K*α radiation
μ = 0.24 mm^−1^
*T* = 100 K
0.77 × 0.21 × 0.11 mm

#### Data collection

Bruker SMART APEXII CCD area-detector diffractometer
Absorption correction: multi-scan (*SADABS*; Bruker, 2009 ▶) *T*_min_ = 0.837, *T*_max_ = 0.974
10178 measured reflections
3646 independent reflections
3596 reflections with *I* > 2σ(*I*)
*R*_int_ = 0.020

#### Refinement

*R*[*F* ^2^ > 2σ(*F* ^2^)] = 0.024
*wR*(*F* ^2^) = 0.062
*S* = 1.05
3646 reflections
253 parameters
3 restraints
H-atom parameters constrained
Δρ_max_ = 0.17 e Å^−3^
Δρ_min_ = −0.22 e Å^−3^
Absolute structure: Flack (1983 ▶), 1556 Friedel pairs
Flack parameter: 0.06 (3)

### 

Data collection: *APEX2* (Bruker, 2009 ▶); cell refinement: *SAINT* (Bruker, 2009 ▶); data reduction: *SAINT*; program(s) used to solve structure: *SHELXTL* (Sheldrick, 2008 ▶); program(s) used to refine structure: *SHELXTL*; molecular graphics: *SHELXTL*; software used to prepare material for publication: *SHELXTL* and *PLATON* (Spek, 2009 ▶).

## Supplementary Material

Crystal structure: contains datablocks global, I. DOI: 10.1107/S1600536810047215/is2632sup1.cif

Structure factors: contains datablocks I. DOI: 10.1107/S1600536810047215/is2632Isup2.hkl

Additional supplementary materials:  crystallographic information; 3D view; checkCIF report

## Figures and Tables

**Table 1:** Hydrogen-bond geometry (Å, °)

*D*—H⋯*A*	*D*—H	H⋯*A*	*D*⋯*A*	*D*—H⋯*A*
C2—H2*A*⋯O1^i^	0.93	2.38	3.1196 (19)	136
C7—H7*A*⋯F1^ii^	0.93	2.50	3.2099 (15)	133
C14—H14*A*⋯F1^ii^	0.93	2.41	3.2614 (17)	153
C17—H17*B*⋯N2	0.97	2.16	2.9091 (18)	133

Symmetry codes: (i) 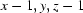; (ii) 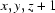.

**Fig. 1.**
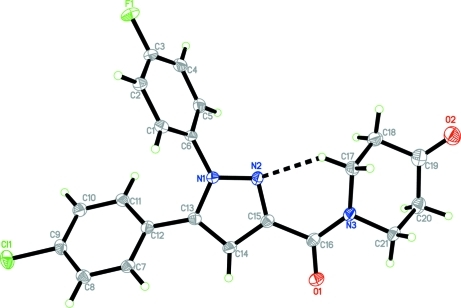
The molecular structure of the title compound showing 50% probability displacement ellipsoids and the atom numbering scheme. Intermolecular hydrogen boding (dashed lines) are omitted for clarity.

**Fig. 2.**
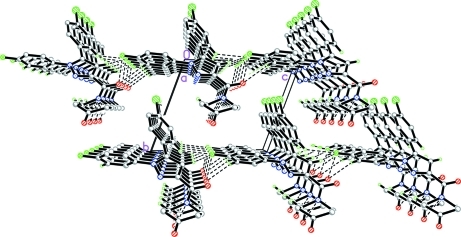
The crystal packing of the title compound viewed along a axis. Intermolecular hydrogen bonds linked the molecules into two-dimensional sheets parallel to the ac plane.

## supplementary crystallographic information

### Comment

Antibacterial and antifungal activities of the azoles are most widely studied and some of them are in clinical practice as anti-microbial agents. However, the azole-resistant strain had led to the development of new antimicrobial compounds. In particular, pyrazole derivatives are extensively studied and used as antimicrobial agents. Pyrazole is an important class of heterocyclic compounds and many pyrazole derivatives are reported to have the broad spectrum of biological properties such as anti-inflammatory, antifungal, herbicidal, anti-tumour, cytotoxic, molecular modelling and antiviral activities. Pyrazole derivatives also act as antiangiogenic agents, A3 adenosine receptor antagonists, neuropeptide YY5 receptor antagonists, kinase inhibitor for treatment of type 2 diabetes, hyperlipidemia, obesity and thrombopiotinmimetics. Recently urea derivatives of pyrazoles have been reported as potent inhibitors of p38 kinase. Since the high electronegativity of halogens (particularly chlorine and fluorine) in the aromatic part of the drug molecules play an important role in enhancing their biological activity, we are interested to have 4-fluoro or 4-chloro substitution in the aryls of 1,5-diaryl pyrazoles. As part of our on-going research aiming the synthesis of new antimicrobial compounds, we have reported the synthesis of novel pyrazole derivatives and their microbial activities (Ragavan *et al.*, 2009; 2010). The structure of the title compound is presented here.

The asymmetric unit of the title compound (Fig. 1), consists of four rings, namely chlorophenyl (C7–C12/Cl1), 4-oxopiperidine-1-carbaldehyde (C16–C21/N3/O1/O2), fluorophenyl(C1–C6/F1) and 1*H*-pyrazole (N1/N2/C13–C15) rings. The 1*H*-pyrazole ring is essentially planar [maximum deviation of 0.002 (1) Å at atoms C13 and C15] and makes dihedral angles of 36.73 (7), 18.73 (7) and 60.88 (8)°, with the chlorophenyl [maximum deviation of 0.0077 (4) Å at atom Cl1], fluorophenyl [maximum deviation of 0.0084 (14) Å at atom C6] and 4-oxopiperidine-1-carbaldehyde [with the r.m.s. deviation of 0.3007 (15) Å] rings. Bond lengths (Allen *et al.*, 1987) and angles are normal and comparable to the related structure (Shahani *et al.*, 2010). The molecular structure is stabilized by an intramolecular C17—H17B···N2 hydrogen bond which forms an *S*(6) ring motif.

In the crystal packing (Fig. 2), intermolecular C14—H14A···F1^ii^and C7—H7A···F1^ii^ hydrogen bonds (Table 1) connect the neighbouring molecules, generating an *R*_2_^1^(7) ring motif. Intermolecular C2—H2A···O1^i^ hydrogen bonds (Table 1) further link the molecules into two-dimensional sheets parallel to the *ac* plane.

### Experimental

The compound has been synthesized using the method available in the literature (Ragavan *et al.*, 2009) and recrystallized using the methanol-chloroform (1:1) mixture (yield 76%, m.p. 436.2–437.5 K).

### Refinement

H atoms were positioned geometrically (C—H = 0.93 or 0.97 Å) and refined using a riding model, with *U*_iso_(H) = 1.2*U*_eq_(C).

### Crystal data

**Table:**

C_21_H_17_ClFN_3_O_2_	*Z* = 1
*M**_r_* = 397.83	*F*(000) = 206
Triclinic, *P*1	*D*_x_ = 1.430 Mg m^−^^3^
Hall symbol: P 1	Mo *K*α radiation, λ = 0.71073 Å
*a* = 6.0341 (2) Å	Cell parameters from 9510 reflections
*b* = 8.2500 (3) Å	θ = 2.2–35.0°
*c* = 10.2448 (3) Å	µ = 0.24 mm^−^^1^
α = 108.837 (1)°	*T* = 100 K
β = 104.782 (1)°	Needle, colourless
γ = 92.792 (1)°	0.77 × 0.21 × 0.11 mm
*V* = 461.90 (3) Å^3^	

### Data collection

**Table:**

Bruker SMART APEXII CCD area-detector diffractometer	3646 independent reflections
Radiation source: fine-focus sealed tube	3596 reflections with *I* > 2σ(*I*)
graphite	*R*_int_ = 0.020
φ and ω scans	θ_max_ = 27.5°, θ_min_ = 2.2°
Absorption correction: multi-scan (*SADABS*; Bruker, 2009)	*h* = −7→7
*T*_min_ = 0.837, *T*_max_ = 0.974	*k* = −10→10
10178 measured reflections	*l* = −13→13

### Refinement

**Table:**

Refinement on *F*^2^	Secondary atom site location: difference Fourier map
Least-squares matrix: full	Hydrogen site location: inferred from neighbouring sites
*R*[*F*^2^ > 2σ(*F*^2^)] = 0.024	H-atom parameters constrained
*wR*(*F*^2^) = 0.062	*w* = 1/[σ^2^(*F*_o_^2^) + (0.0375*P*)^2^ + 0.0616*P*] where *P* = (*F*_o_^2^ + 2*F*_c_^2^)/3
*S* = 1.05	(Δ/σ)_max_ = 0.001
3646 reflections	Δρ_max_ = 0.17 e Å^−^^3^
253 parameters	Δρ_min_ = −0.21 e Å^−^^3^
3 restraints	Absolute structure: Flack (1983), 1556 Friedel pairs
Primary atom site location: structure-invariant direct methods	Flack parameter: 0.06 (3)

### Special details

**Table:**

Experimental. The crystal was placed in the cold stream of an Oxford Cyrosystems Cobra open-flow nitrogen cryostat (Cosier & Glazer, 1986) operating at 100.0 (1) K.
Geometry. All e.s.d.'s (except the e.s.d. in the dihedral angle between two l.s. planes) are estimated using the full covariance matrix. The cell e.s.d.'s are taken into account individually in the estimation of e.s.d.'s in distances, angles and torsion angles; correlations between e.s.d.'s in cell parameters are only used when they are defined by crystal symmetry. An approximate (isotropic) treatment of cell e.s.d.'s is used for estimating e.s.d.'s involving l.s. planes.
Refinement. Refinement of *F*^2^ against ALL reflections. The weighted *R*-factor *wR* and goodness of fit *S* are based on *F*^2^, conventional *R*-factors *R* are based on *F*, with *F* set to zero for negative *F*^2^. The threshold expression of *F*^2^ > σ(*F*^2^) is used only for calculating *R*-factors(gt) *etc*. and is not relevant to the choice of reflections for refinement. *R*-factors based on *F*^2^ are statistically about twice as large as those based on *F*, and *R*- factors based on ALL data will be even larger.

### Fractional atomic coordinates and isotropic or equivalent isotropic displacement parameters (Å^2^)

**Table:**

	*x*	*y*	*z*	*U*_iso_*/*U*_eq_	
Cl1	−0.18751 (5)	−0.56629 (4)	−0.09987 (4)	0.02699 (9)	
F1	0.47475 (18)	−0.01865 (15)	−0.50052 (9)	0.0397 (3)	
O1	1.11635 (17)	0.26727 (13)	0.53957 (10)	0.0225 (2)	
O2	1.85032 (19)	0.66828 (16)	0.41014 (12)	0.0326 (3)	
N1	0.73866 (19)	0.06297 (14)	0.07455 (11)	0.0160 (2)	
N2	0.92725 (19)	0.18040 (14)	0.16028 (11)	0.0171 (2)	
N3	1.27132 (19)	0.41856 (15)	0.42751 (11)	0.0197 (2)	
C1	0.4556 (2)	0.08181 (18)	−0.13442 (14)	0.0192 (3)	
H1A	0.3585	0.1228	−0.0774	0.023*	
C2	0.3886 (3)	0.06260 (19)	−0.27928 (14)	0.0235 (3)	
H2A	0.2462	0.0894	−0.3215	0.028*	
C3	0.5399 (3)	0.00258 (19)	−0.35841 (14)	0.0254 (3)	
C4	0.7542 (3)	−0.03780 (19)	−0.30190 (14)	0.0243 (3)	
H4A	0.8525	−0.0754	−0.3587	0.029*	
C5	0.8185 (2)	−0.02049 (18)	−0.15695 (14)	0.0200 (3)	
H5A	0.9602	−0.0487	−0.1154	0.024*	
C6	0.6687 (2)	0.03934 (16)	−0.07542 (13)	0.0161 (2)	
C7	0.2588 (2)	−0.14175 (17)	0.15655 (13)	0.0173 (3)	
H7A	0.2749	−0.0537	0.2437	0.021*	
C8	0.0689 (2)	−0.27130 (18)	0.10073 (14)	0.0190 (3)	
H8A	−0.0413	−0.2705	0.1498	0.023*	
C9	0.0469 (2)	−0.40181 (17)	−0.02954 (15)	0.0192 (3)	
C10	0.2091 (3)	−0.40594 (18)	−0.10451 (15)	0.0199 (3)	
H10A	0.1914	−0.4943	−0.1918	0.024*	
C11	0.3994 (2)	−0.27583 (17)	−0.04738 (14)	0.0188 (3)	
H11A	0.5098	−0.2778	−0.0966	0.023*	
C12	0.4254 (2)	−0.14175 (16)	0.08395 (13)	0.0160 (3)	
C13	0.6292 (2)	−0.00678 (16)	0.15007 (13)	0.0151 (2)	
C14	0.7564 (2)	0.07058 (17)	0.29276 (13)	0.0172 (3)	
H14A	0.7284	0.0510	0.3719	0.021*	
C15	0.9368 (2)	0.18496 (17)	0.29308 (13)	0.0164 (3)	
C16	1.1168 (2)	0.29506 (17)	0.42851 (13)	0.0168 (3)	
C17	1.2640 (2)	0.49206 (18)	0.31390 (14)	0.0202 (3)	
H17A	1.2384	0.6119	0.3472	0.024*	
H17B	1.1365	0.4287	0.2296	0.024*	
C18	1.4914 (3)	0.48167 (19)	0.27422 (15)	0.0225 (3)	
H18A	1.5073	0.3614	0.2298	0.027*	
H18B	1.4901	0.5391	0.2051	0.027*	
C19	1.6952 (3)	0.56654 (19)	0.40686 (15)	0.0240 (3)	
C20	1.6883 (2)	0.5158 (2)	0.53511 (15)	0.0239 (3)	
H20A	1.8015	0.5955	0.6202	0.029*	
H20B	1.7320	0.4010	0.5200	0.029*	
C21	1.4500 (2)	0.51622 (19)	0.56187 (14)	0.0203 (3)	
H21A	1.4476	0.4642	0.6341	0.024*	
H21B	1.4190	0.6344	0.5975	0.024*	

### Atomic displacement parameters (Å^2^)

**Table:**

	*U*^11^	*U*^22^	*U*^33^	*U*^12^	*U*^13^	*U*^23^
Cl1	0.02313 (16)	0.01987 (16)	0.03408 (18)	−0.00462 (12)	0.00415 (13)	0.00849 (13)
F1	0.0393 (5)	0.0625 (7)	0.0143 (4)	−0.0089 (5)	0.0024 (4)	0.0159 (4)
O1	0.0247 (5)	0.0267 (5)	0.0153 (4)	0.0003 (4)	0.0023 (4)	0.0092 (4)
O2	0.0231 (5)	0.0389 (6)	0.0328 (6)	−0.0016 (5)	0.0122 (4)	0.0060 (5)
N1	0.0171 (5)	0.0171 (5)	0.0133 (5)	−0.0006 (4)	0.0035 (4)	0.0058 (4)
N2	0.0162 (5)	0.0173 (5)	0.0150 (5)	−0.0008 (4)	0.0013 (4)	0.0048 (4)
N3	0.0203 (6)	0.0210 (6)	0.0139 (5)	−0.0026 (5)	−0.0014 (4)	0.0065 (4)
C1	0.0194 (6)	0.0214 (7)	0.0187 (6)	−0.0005 (5)	0.0056 (5)	0.0097 (5)
C2	0.0222 (7)	0.0291 (8)	0.0196 (7)	−0.0038 (6)	0.0001 (5)	0.0145 (6)
C3	0.0308 (7)	0.0295 (8)	0.0129 (6)	−0.0090 (6)	0.0024 (5)	0.0085 (5)
C4	0.0281 (7)	0.0243 (7)	0.0183 (6)	−0.0055 (6)	0.0098 (6)	0.0033 (5)
C5	0.0199 (6)	0.0182 (6)	0.0196 (6)	−0.0016 (5)	0.0057 (5)	0.0041 (5)
C6	0.0197 (6)	0.0160 (6)	0.0127 (5)	−0.0023 (5)	0.0040 (5)	0.0062 (4)
C7	0.0203 (6)	0.0173 (6)	0.0151 (6)	0.0034 (5)	0.0039 (5)	0.0075 (5)
C8	0.0187 (6)	0.0213 (7)	0.0206 (6)	0.0038 (5)	0.0061 (5)	0.0114 (5)
C9	0.0175 (6)	0.0159 (6)	0.0244 (7)	−0.0009 (5)	0.0019 (5)	0.0109 (5)
C10	0.0240 (7)	0.0165 (6)	0.0182 (6)	0.0011 (5)	0.0051 (5)	0.0055 (5)
C11	0.0211 (6)	0.0174 (6)	0.0184 (6)	0.0012 (5)	0.0062 (5)	0.0068 (5)
C12	0.0179 (6)	0.0149 (6)	0.0155 (5)	0.0021 (5)	0.0017 (5)	0.0081 (5)
C13	0.0174 (6)	0.0142 (6)	0.0154 (5)	0.0035 (5)	0.0055 (5)	0.0065 (5)
C14	0.0206 (6)	0.0178 (6)	0.0138 (6)	0.0028 (5)	0.0045 (5)	0.0065 (5)
C15	0.0182 (6)	0.0161 (6)	0.0147 (6)	0.0035 (5)	0.0031 (5)	0.0060 (5)
C16	0.0172 (6)	0.0172 (6)	0.0151 (6)	0.0044 (5)	0.0027 (5)	0.0055 (5)
C17	0.0202 (6)	0.0202 (6)	0.0195 (6)	−0.0005 (5)	0.0022 (5)	0.0090 (5)
C18	0.0258 (7)	0.0229 (7)	0.0192 (6)	0.0035 (6)	0.0074 (5)	0.0070 (5)
C19	0.0196 (7)	0.0237 (7)	0.0260 (7)	0.0062 (6)	0.0086 (5)	0.0033 (6)
C20	0.0190 (7)	0.0258 (7)	0.0218 (7)	0.0029 (6)	0.0021 (5)	0.0047 (5)
C21	0.0192 (6)	0.0224 (7)	0.0144 (6)	−0.0004 (5)	0.0011 (5)	0.0032 (5)

### Geometric parameters (Å, °)

**Table:**

Cl1—C9	1.7387 (14)	C8—C9	1.3877 (19)
F1—C3	1.3570 (14)	C8—H8A	0.9300
O1—C16	1.2317 (16)	C9—C10	1.385 (2)
O2—C19	1.2130 (19)	C10—C11	1.394 (2)
N1—N2	1.3558 (15)	C10—H10A	0.9300
N1—C13	1.3701 (17)	C11—C12	1.4045 (18)
N1—C6	1.4299 (15)	C11—H11A	0.9300
N2—C15	1.3352 (16)	C12—C13	1.4714 (18)
N3—C16	1.3515 (18)	C13—C14	1.3823 (17)
N3—C21	1.4657 (15)	C14—C15	1.4039 (19)
N3—C17	1.4684 (17)	C14—H14A	0.9300
C1—C6	1.3875 (19)	C15—C16	1.5004 (17)
C1—C2	1.3881 (18)	C17—C18	1.526 (2)
C1—H1A	0.9300	C17—H17A	0.9700
C2—C3	1.378 (2)	C17—H17B	0.9700
C2—H2A	0.9300	C18—C19	1.516 (2)
C3—C4	1.379 (2)	C18—H18A	0.9700
C4—C5	1.3927 (18)	C18—H18B	0.9700
C4—H4A	0.9300	C19—C20	1.511 (2)
C5—C6	1.3862 (18)	C20—C21	1.531 (2)
C5—H5A	0.9300	C20—H20A	0.9700
C7—C8	1.3903 (19)	C20—H20B	0.9700
C7—C12	1.3951 (19)	C21—H21A	0.9700
C7—H7A	0.9300	C21—H21B	0.9700
			
N2—N1—C13	112.88 (10)	C7—C12—C13	119.32 (11)
N2—N1—C6	118.05 (11)	C11—C12—C13	121.72 (12)
C13—N1—C6	128.81 (11)	N1—C13—C14	105.49 (12)
C15—N2—N1	104.32 (11)	N1—C13—C12	124.42 (11)
C16—N3—C21	118.56 (11)	C14—C13—C12	130.04 (12)
C16—N3—C17	128.10 (11)	C13—C14—C15	105.57 (12)
C21—N3—C17	112.47 (11)	C13—C14—H14A	127.2
C6—C1—C2	119.43 (13)	C15—C14—H14A	127.2
C6—C1—H1A	120.3	N2—C15—C14	111.75 (11)
C2—C1—H1A	120.3	N2—C15—C16	125.60 (12)
C3—C2—C1	117.83 (13)	C14—C15—C16	122.64 (11)
C3—C2—H2A	121.1	O1—C16—N3	122.03 (11)
C1—C2—H2A	121.1	O1—C16—C15	116.83 (12)
F1—C3—C2	118.27 (13)	N3—C16—C15	121.13 (11)
F1—C3—C4	117.82 (13)	N3—C17—C18	110.09 (11)
C2—C3—C4	123.90 (12)	N3—C17—H17A	109.6
C3—C4—C5	117.84 (13)	C18—C17—H17A	109.6
C3—C4—H4A	121.1	N3—C17—H17B	109.6
C5—C4—H4A	121.1	C18—C17—H17B	109.6
C6—C5—C4	119.19 (12)	H17A—C17—H17B	108.2
C6—C5—H5A	120.4	C19—C18—C17	110.61 (11)
C4—C5—H5A	120.4	C19—C18—H18A	109.5
C5—C6—C1	121.79 (12)	C17—C18—H18A	109.5
C5—C6—N1	119.07 (11)	C19—C18—H18B	109.5
C1—C6—N1	119.11 (12)	C17—C18—H18B	109.5
C8—C7—C12	121.09 (12)	H18A—C18—H18B	108.1
C8—C7—H7A	119.5	O2—C19—C20	122.68 (13)
C12—C7—H7A	119.5	O2—C19—C18	122.62 (14)
C9—C8—C7	118.83 (13)	C20—C19—C18	114.69 (13)
C9—C8—H8A	120.6	C19—C20—C21	113.13 (11)
C7—C8—H8A	120.6	C19—C20—H20A	109.0
C10—C9—C8	121.64 (13)	C21—C20—H20A	109.0
C10—C9—Cl1	118.91 (10)	C19—C20—H20B	109.0
C8—C9—Cl1	119.45 (11)	C21—C20—H20B	109.0
C9—C10—C11	119.11 (12)	H20A—C20—H20B	107.8
C9—C10—H10A	120.4	N3—C21—C20	109.72 (11)
C11—C10—H10A	120.4	N3—C21—H21A	109.7
C10—C11—C12	120.45 (13)	C20—C21—H21A	109.7
C10—C11—H11A	119.8	N3—C21—H21B	109.7
C12—C11—H11A	119.8	C20—C21—H21B	109.7
C7—C12—C11	118.89 (13)	H21A—C21—H21B	108.2
			
C13—N1—N2—C15	−0.07 (13)	C6—N1—C13—C12	8.4 (2)
C6—N1—N2—C15	174.57 (11)	C7—C12—C13—N1	−146.36 (12)
C6—C1—C2—C3	−0.5 (2)	C11—C12—C13—N1	36.75 (19)
C1—C2—C3—F1	179.21 (13)	C7—C12—C13—C14	36.9 (2)
C1—C2—C3—C4	−0.6 (2)	C11—C12—C13—C14	−140.04 (14)
F1—C3—C4—C5	−178.30 (12)	N1—C13—C14—C15	0.40 (14)
C2—C3—C4—C5	1.5 (2)	C12—C13—C14—C15	177.65 (12)
C3—C4—C5—C6	−1.3 (2)	N1—N2—C15—C14	0.34 (14)
C4—C5—C6—C1	0.3 (2)	N1—N2—C15—C16	179.57 (11)
C4—C5—C6—N1	−177.75 (12)	C13—C14—C15—N2	−0.47 (15)
C2—C1—C6—C5	0.6 (2)	C13—C14—C15—C16	−179.73 (11)
C2—C1—C6—N1	178.67 (12)	C21—N3—C16—O1	2.92 (18)
N2—N1—C6—C5	62.36 (15)	C17—N3—C16—O1	−165.56 (13)
C13—N1—C6—C5	−123.97 (15)	C21—N3—C16—C15	−177.10 (11)
N2—N1—C6—C1	−115.72 (14)	C17—N3—C16—C15	14.4 (2)
C13—N1—C6—C1	57.94 (19)	N2—C15—C16—O1	−171.13 (13)
C12—C7—C8—C9	−0.09 (18)	C14—C15—C16—O1	8.02 (18)
C7—C8—C9—C10	0.12 (19)	N2—C15—C16—N3	8.88 (19)
C7—C8—C9—Cl1	179.22 (10)	C14—C15—C16—N3	−171.97 (12)
C8—C9—C10—C11	0.13 (19)	C16—N3—C17—C18	−127.53 (14)
Cl1—C9—C10—C11	−178.98 (10)	C21—N3—C17—C18	63.42 (14)
C9—C10—C11—C12	−0.41 (19)	N3—C17—C18—C19	−54.44 (15)
C8—C7—C12—C11	−0.19 (18)	C17—C18—C19—O2	−132.83 (14)
C8—C7—C12—C13	−177.17 (11)	C17—C18—C19—C20	46.56 (16)
C10—C11—C12—C7	0.44 (19)	O2—C19—C20—C21	134.59 (15)
C10—C11—C12—C13	177.34 (12)	C18—C19—C20—C21	−44.80 (16)
N2—N1—C13—C14	−0.21 (14)	C16—N3—C21—C20	129.76 (13)
C6—N1—C13—C14	−174.15 (12)	C17—N3—C21—C20	−60.04 (15)
N2—N1—C13—C12	−177.66 (11)	C19—C20—C21—N3	49.66 (16)

### Hydrogen-bond geometry (Å, °)

**Table:**

*D*—H···*A*	*D*—H	H···*A*	*D*···*A*	*D*—H···*A*
C2—H2A···O1^i^	0.93	2.38	3.1196 (19)	136
C7—H7A···F1^ii^	0.93	2.50	3.2099 (15)	133
C14—H14A···F1^ii^	0.93	2.41	3.2614 (17)	153
C17—H17B···N2	0.97	2.16	2.9091 (18)	133

Symmetry codes: (i) *x*−1, *y*, *z*−1; (ii) *x*, *y*, *z*+1.
